# Comprehensive analysis of mitochondrial energy metabolism–related genes and immune infiltration in intervertebral disk degeneration

**DOI:** 10.1097/MD.0000000000044306

**Published:** 2025-09-05

**Authors:** Jianlan Lv, Zhenwei Wang

**Affiliations:** a Department of Rehabilitation Medicine, People’s Hospital of Anji, Huzhou, Zhejiang Province, China; b The Third School of Clinical Medicine, Zhejiang Chinese Medical University, Hangzhou, China.

**Keywords:** immune infiltration, intervertebral disc degeneration, key genes, mitochondrial energy metabolism

## Abstract

Lower back pain caused by intervertebral disk degeneration (IDD) is a common problem among middle-aged and older adults. We aimed to identify novel diagnostic biomarkers of IDD and analyze the potential association between key genes and immune cell infiltration. We screened differentially expressed genes (DEGs) related to IDD and gene sets associated with mitochondrial energy metabolism using the Gene Expression Omnibus and GeneCards databases, respectively. Subsequently, we used multiple enrichment analysis methods to determine the biological functionalities of mitochondrial energy metabolism–related differentially expressed genes (MEMRDEGs). Key genes were selected using logistic regression analysis, a support vector machine algorithm, and least absolute shrinkage and selection operator regression analysis to construct an IDD diagnostic model. To obtain further insights, we examined the relationship between key genes and the presence of infiltrating immune cells. We screened 1304 DEGs that exhibited substantial differences in 20 pathways, including the Wnt signaling pathway, between the IDD and control groups. We identified 33 MEMRDEGs and selected 7 key genes (*NDUFA6*, *YWHAZ*, *DLAT*, *BDNF*, *ECI2*, *ACO1*, and *ALDH7A1*) to construct an IDD diagnostic model. Receiver operating characteristic curve analysis revealed that these genes exhibited high accuracy in assessing IDD risk, with *BDNF* and *DLAT* particularly distinguishing between the low- and high-risk IDD groups. Finally, using single-sample gene set enrichment analysis, we identified a relationship between IDD and immune infiltration, with most immune cells showing strong correlations. A significant positive relationship was found between *ACO1* and the immune cells, known as immature dendritic cells. These results offer remarkable insights into the mechanisms underlying the occurrence and development of IDD, potentially identifying new opportunities for diagnosis and therapeutic intervention.

## 1. Introduction

Lower back pain (LBP) is a global health issue^[1]^ prevalent among adults. This trend is rapidly intensifying with the seniorization of society and the widespread existence of unhealthy ways of life. Intervertebral disk degeneration (IDD) is a major degenerative condition that is closely associated with LBP^[2]^ and forms the pathological basis of many degenerative spinal disorders. The intervertebral disk is a complex structure comprising the inner nucleus pulposus (NP), peripheral annulus fibrosus (AF), and cartilaginous endplate (CEP). These components work together to maintain the structural and functional integrity of the intervertebral disk. The pathogenesis of IDD is complex and multifaceted, and NP degeneration is a key factor in IDD.^[3]^ As individuals age, the cellular characteristics of the NP cells gradually decrease, leading to the loss of hydrophilic matrix molecules in the extracellular matrix (ECM), which disrupts the biomechanical properties of the intervertebral disk and initiates a series of pathophysiological reactions. Initially, inflammation increases, causing swelling and pain in local tissues. Next, pain factors, including nerve compression and pain mediators produced by inflammatory reactions, also increase. Collectively, these changes contribute to the occurrence and development of LBP.^[4]^ To effectively treat LBP and IDD, it is necessary to understand the pathophysiological mechanisms and develop effective treatment methods targeting these mechanisms. Future research should focus on protecting intervertebral disk cells from damage, promoting regeneration and repair of the ECM, and reducing inflammation and pain.

A study has shown that mitochondrion-related genes play a central role in cellular function by regulating the immune response of cells, particularly when NP cells are damaged.^[5]^ Mitochondrial energy metabolism primarily involves the conversion of nutrients (sugars, fats, and proteins) into adenosine triphosphate (ATP). Genetic abnormalities in mitochondrial energy metabolism pathways, such as abnormal expression or mutations, can lead to impairments in the energy conversion process, subsequently affecting the normal function of cells. These abnormalities have potential links to various diseases (such as neurodegenerative diseases and metabolic diseases), inflammation, and tumors,^[6]^ and immune cells play a crucial role in these processes. To summarize, immune cell infiltration and mitochondrial energy metabolism significantly contribute to the pathogenesis and development of IDD. Nevertheless, the precise mechanisms of mitochondrial energy metabolism in IDD and its relationship with immune cell infiltration remain to be elucidated.

Research has shown that immune cell infiltration has a significant effect on the pathogenesis and development of IDD.^[7,8]^ Immune cells accelerate the inflammatory cascade by releasing inflammatory mediators and increasing oxidative stress, which further disrupts internal homeostasis of the intervertebral disk and triggers an immune response.^[9,10]^ The NP, as an immune-privileged organ, becomes exposed to an immune attack once its isolation from the immunological system via the AF and CEP is disrupted.^[11]^ Due to the uncommon tissue composition within the intervertebral disk, its degenerative process exhibits a unique immune cell infiltration arrangement, providing new immune targets for the treatment of IDD.^[10]^

In this study, we aimed to identify the mitochondrial energy metabolism–related genes (MEMRGs) that are involved in the process of IDD and determine which biological functions are associated with the progression of IDD. Based on high-throughput sequencing datasets of patients with IDD from public databases, we conducted an in-depth bioinformatics analysis. By comprehensively applying various computational methods and statistical approaches, we performed an extensive comparative analysis of genes in the intervertebral disks of healthy individuals and patients with IDD. This study aims to clarify the etiology of IDD and explore new avenues for precise therapeutic interventions in cases of LBP.

## 2. Materials and methods

### 2.1. Data collection

Datasets GSE34095 and GSE147383^[12]^ of IDD were gathered from the Gene Expression Omnibus (GEO) (https://www.ncbi.nlm.nih.gov/geo/) using the R package GEO query.^[13]^ The chip platforms GSE34095 and GSE147383 are GPL96 and GPL570, respectively. Specific information is shown in Table 1. The GSE34095 dataset contained 3 IDD and 3 control samples, whereas the GSE147383 dataset contained 4 IDD and 4 standard samples. All IDD and standard samples included in this study were obtained from the IDD tissue of Homo sapiens.

**Table 1:** GEO microarray chip information.

	GSE34095	GSE147383
Platform	GPL96	GPL570
Type	Array	Array
Species	Homo sapiens	Homo sapiens
Tissue	Intervertebral disc	Intervertebral disc
Samples in IDD group	3	4
Samples in control group	3	4
Reference	/	PMID: 33382035

GEO = Gene Expression Omnibus, IDD = intervertebral disk degeneration.

We collected MEMRGs using the GeneCards database^[14]^ (https://www.genecards.org/) and published literature. The GeneCards database provides comprehensive information on human genes. We utilized “Mitochondrial Energy Metabolism” as a search keyword and kept exclusively “Protein Coding” and “Relevance Score > 0” genes, a total of 220 MEMRGs were obtained. Additionally, we searched the PubMed website (https://pubmed.ncbi.nlm.nih.gov/) with the keyword “Mitochondrial Energy Metabolism” to get published literature^[15]^ on MEMRGs. In total, 381 MEMRGs were obtained after the combined duplication removal. The details are shown in Supplementary 1 (Supplemental Digital Content, https://links.lww.com/MD/P866). Finally, the R package limma^[16]^ was used to regularize the combined GEO datasets, annotate the probes, and standardize them. The pre- and postcorrection expression matrices, where the correction addresses the batch effect, were subjected to principal component analysis (PCA)^[17]^ to appraise the success of batch effect elimination. PCA is a data-dimensionality reduction approach that identifies feature vectors (components) within high-dimensional data.

### 2.2. Determination of energy metabolism–related differentially expressed genes (MEMRDEGs)

In the merged datasets, the samples were allocated to either the IDD or control group. Differential gene expression analysis was conducted between the IDD and control groups using the R package limma, and the threshold for differentially expressed genes (DEGs) was set as |logFC| > 0 and *P *< .05 to include more DEGs to ensure that changes that may be biologically significant in disease progression were not missed. Among them, genes with logFC > 0 and *P*-value < .05 were DEGs with upregulated expressions, and genes with logFC < 0 and *P*-value < .05 were DEGs with downregulated expressions. The results of the differential analysis were plotted using the R package ggplot2 volcano plot. To identify MEMRDEGs associated with IDD, we integrated the GEO dataset to obtain genes that were differentially expressed in association with MEMRGs using variance analysis with the standards of |logFC| > 0 and *P* < .05 and then intersected the genes with the Venn map to identify the MEMRDEGs. Utilizing the R package pheatmap, heat maps for the MEMRDEGs were produced.

### 2.3. Differential expression and correlation analysis of MEMRDEGs

To investigate the disparate expression of MEMRDEGs between the IDD and control groups, a comparative map for the groups was created based on the expression of MEMRDEGs. To examine the relationship between MEMRDEGs in-depth, the correlation of their expression across aggregated datasets was measured using the Spearman algorithm. The outcomes of the correlation analysis were visually displayed using a correlation heatmap generated with the R package pheatmap. Subsequently, the MEMRDEGs with the strongest positive and negative correlations were identified, and a scatter plot was designed using the ggplot2 R package to visualize and analyze the correlations for clear representation. The absolute value of the correlation coefficient (*R*-value) below 0.3 was weak or no correlation, 0.3 to 0.5 was a weak correlation, 0.5 to 0.8 was a moderate correlation, and above 0.8 was a strong correlation.

### 2.4. Gene set enrichment analysis (GSEA)

Gene set enrichment analysis (GSEA)^[18]^ was applied to determine the dispersal pattern of genes in a defined set, within a table of genes sorted by their correlation with phenotypic traits. This investigation helped determine the contribution of these genes to the observable characteristics. Genes from the combined GEO datasets were ranked based on their logFC values. Subsequently, the R package clusterProfiler was used to conduct GSEA for all genes within the integrated GEO datasets. The parameters used in the GSEA were as follows: the seed value was set to 2023, and the minimum and maximum numbers of genes in each gene set were 10 and 500, respectively. Molecular Signatures Database (MSigDB) (https://www.gsea-msigdb.org/gsea/msigdb) was used to access c2 gene sets. (Cp. All. V2022.1. Hs. Symbols). GMT (All Canonical Pathways) (3050) was used to perform GSEA, and the screening criterion for GSEA was *P*-value < .05.

### 2.5. Gene set variation analysis (GSVA)

GSVA was suitable for multi-sample comparisons, which do not take into account the specific ordering of genes, but instead directly calculate the enrichment score of a gene set in a sample, thus measuring the change in activity of a gene pathway between different samples. GSVA,^[19]^ a nonparametric unsupervised method, evaluates gene set enrichment by converting the gene expression profiles of individual genes across samples into a representative matrix of gene sets using microarray data. This permitted the evaluation of whether distinct pathways were prevalent in different samples. Gene sets were obtained from MSigDB in the form c2.cp.v2023.2. Hs.symbols.gmt and GSVA were performed on all genes in the integrated GEO datasets to calculate functional enrichment differences between the IDD and control groups. The screening criterion for GSVA was *P*-value < .05.

### 2.6. Gene ontology (GO) and the Kyoto Encyclopedia of Genes and Genomes (KEGG)

GO analysis is a common technique used for extensive functional enrichment investigations, including the analysis of biological processes (BPs), cell components, and molecular functions.^[20]^ KEGG is a renowned database that encompasses comprehensive information on genomes, biological pathways, diseases, and drugs.^[21]^ GO and KEGG pathway enrichment analyses of MEMRDEGs were conducted using the R package clusterProfiler.^[22]^ The results were considered statistically significant if the *P*-value was < .05 and the false discovery rate value (*q*-value) < 0.25. The *P*-value was corrected by the Benjamini–Hochberg method.

### 2.7. Diagnostic model construction

To construct diagnostic models for IDD using combined GEO datasets, logistic regression analysis was performed on the MEMRDEGs. When the dependent variable was binary, such as the IDD or control groups, logistic regression was employed used to assess the relationship between the independent and dependent variables. A *P*-value < .10 was used as the criterion to identify MEMRDEGs and a logistic regression model was formulated. The forest plot shows the group expression of MEMRDEGs incorporated into the logistic regression model. Subsequently, using the MEMRDEGs encompassed within the logistic regression model, a support vector machine (SVM) algorithm was employed to build the SVM model. Genes with the highest accuracy and lowest error rate were selected as the basis for the model and MEMRDEGs were screened. Lastly, the MEMRDEGs in the SVM model served as input for the least absolute shrinkage and selection operator (LASSO) regression analysis, which was conducted using the glmnet package in R with the specified parameters of the set.seed (500) and family = “binomial.”^[23]^ The LASSO regression analysis was based on linear regression analysis. Adding a penalty term (lambda multiplied by the absolute value of the slope) reduced the overfitting of the model and enhanced its generalizability. Diagnostic model plots and variable trajectory plots were used to graphically illustrate the results of the LASSO regression analysis, which functioned as a diagnostic model for IDD and identified MEMRDEGs as key genes. Finally, the LASSO risk score was computed using the risk coefficient derived from the LASSO analysis. The calculation formula for the risk index was as follows:


$$\begin{array}{l} Risk\;score=\underset{i}{\sum }\;Coefficient\left(gene_{i}\right)*mRNA\;expression\left(gene_{i}\right) \end{array}$$

### 2.8. Diagnostic model validation

We used the R package pROC to draw the receiver operating characteristic (ROC) curve for crucial genes and computed the area under the curve (AUC) to evaluate the predictive accuracy of both the risk score and expression of these genes in predicting IDD. The AUC of the ROC curve was typically between 0.5 and 1, with an AUC between 0.5 and 0.7 indicating low accuracy, an AUC between 0.7 and 0.9 indicating moderate accuracy, and an AUC above 0.9 indicating high accuracy.

The nomogram^[24]^ uses a set of intersecting line segments to represent the functional relationships between multiple covariates in a plane coordinate system. Using the R package rms, a nomogram was generated to visualize the interrelationships between key genes, relying on the outcomes of logistic regression analysis. We plotted a calibration curve to gauge the accuracy and discriminatory ability of the diagnostic model for IDD using data obtained from the LASSO regression analysis. Additionally, using the R package ggDCA and integrating the GEO datasets for key genes, we conducted decision curve analysis (DCA) to plot the DCA graph. The DCA^[25]^ serves as a simple and efficient framework for appraising the utility of clinical prediction models, diagnostic tests, and molecular markers.

### 2.9. Friends analysis

GO annotation provides a quantitative method for calculating the similarity between genes and genomes and has become an important foundation for many bioinformatic analysis methods. We used the GOSemSim package in R^[26]^ to assess the functional similarity between key genes and performed functional similarity (friends) analysis on the functional relationships between key genes.

### 2.10. Regulatory network construction

Transcription factors (TFs) influence gene expression by interacting with significant genes during posttranscriptional processes. We retrieved from the ChIPBase database^[27]^ (https://rnasysu.com/chipbase3/index.php) and filtered based on the number of samples found (upstream + downstream) > 5. We then analyzed how TFs regulate the expressions of essential genes and visualized the regulatory network between mRNAs and TFs using the Cytoscape software. In addition, to explore the relationship between key genes and miRNAs, we accessed the StarBase v3.0, to retrieve relevant miRNAs^[28]^ (https://rnasysu.com/encori/) with the filtering criteria of at least 4 source records for mRNA–miRNA interaction relationships. We then visualized the mRNA–miRNA regulatory network using Cytoscape software. miRNAs exert a crucial regulatory function in biological evolution and development and can modulate numerous target genes, whereas the expression of a single target gene can be regulated by multiple miRNAs.

### 2.11. Immune infiltration analysis

Single-sample gene set enrichment analysis (ssGSEA) was performed to quantify the relative abundance of each immune cell type.^[29]^ First, we annotated various types of infiltrating immune cells. Subsequently, we represented the relative abundance of immune cell infiltration in each sample using the enrichment scores computed by ssGSEA and obtained an immune cell infiltration matrix for the integrated GEO dataset. Using the Spearman algorithm, we calculated the correlation among immune cells, key genes, and immune cells. We generated correlation heat maps and bubble charts to visualize these relationships. With the help of the R packages “pheatmap” and “ggplot2,” we produced heatmaps and bubble charts respectively, to intuitively represent the correlation data among immune cells as well as the linkage between pivotal genes and immune cells.

### 2.12. Statistical analysis

All data processing and analyses in this study were performed using R software (version 4.3.0). To compare 2 continuous variables, the statistical significance of normally distributed variables was evaluated using the independent Student *t* test. Differences between non-normally distributed variables were analyzed using the Mann–Whitney *U* test (Wilcoxon rank-sum test). The Kruskal–Wallis test was used to compare 3 or more groups. The results were calculated using Spearman correlation analysis to obtain the correlation coefficients between distinct molecules. All statistical *P*-values were bilateral, and a *P*-value < .05 was considered statistically significant.

## 3. Results

### 3.1. Data processing

**Figure 1.**
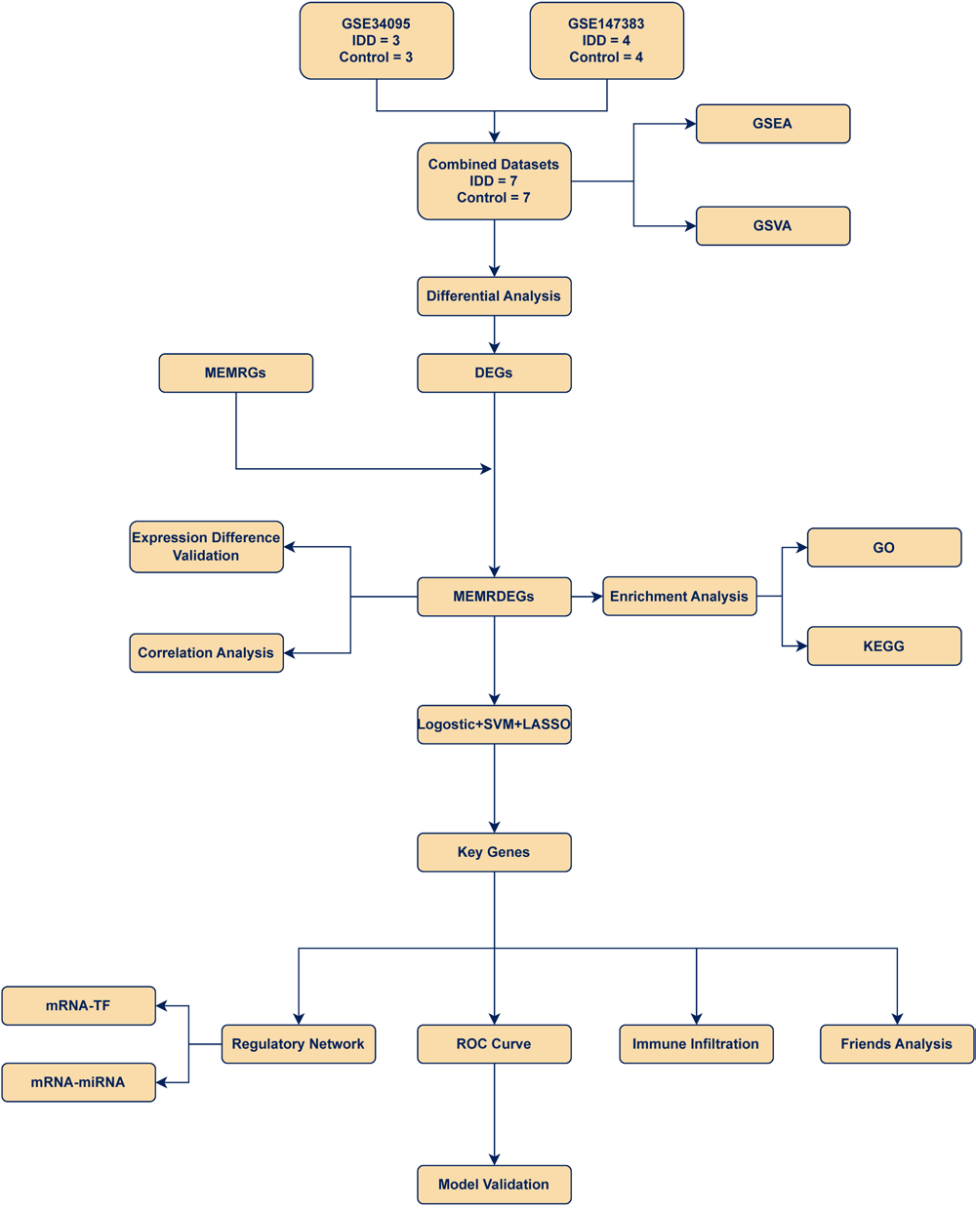
Flow chart for the comprehensive analysis of MEMRDEGs. MEMRDEGs = mitochondrial energy metabolism–related differentially expressed genes.

**Figure 2.**
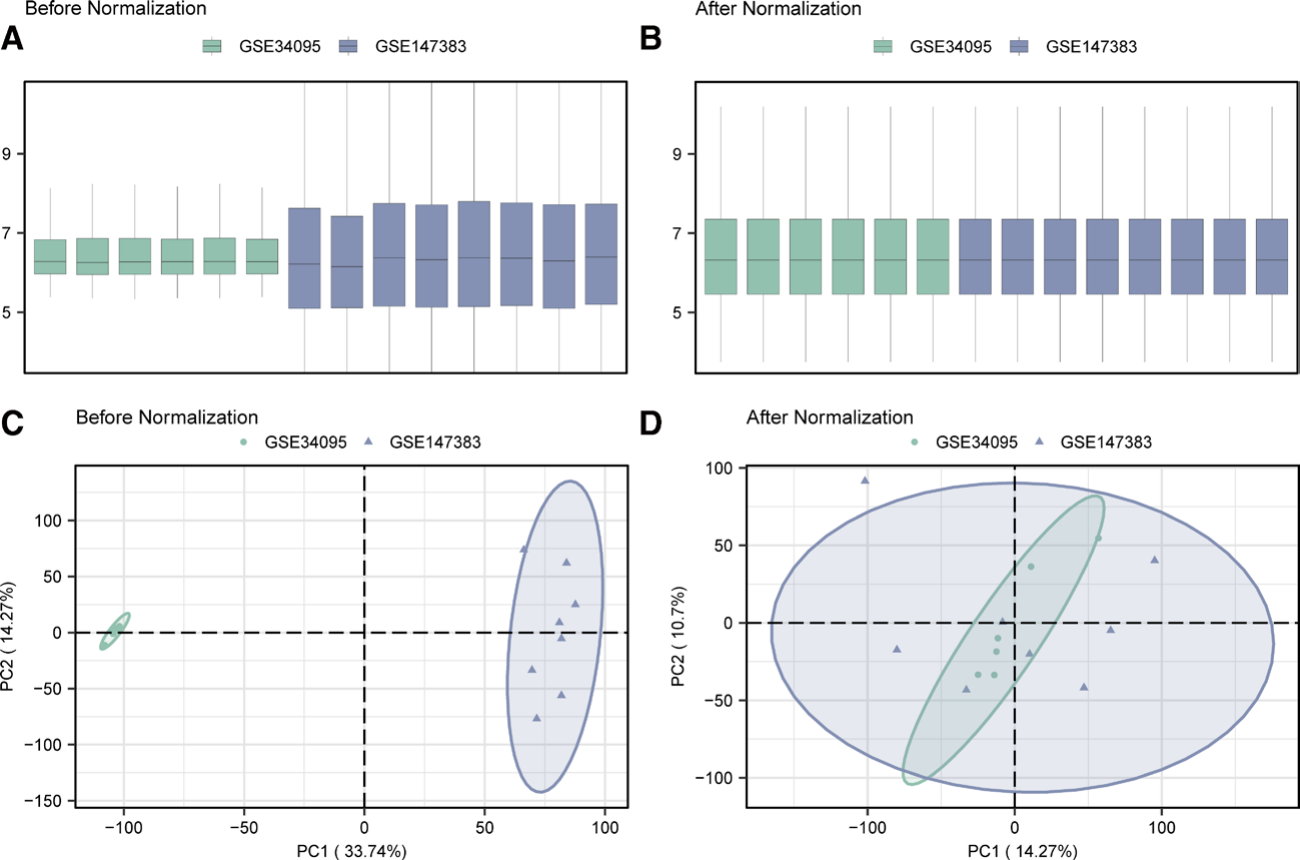
Batch effects removal of GSE34095 and GSE147383. (A) Box plot of combined GEO datasets distribution before-batch removal. (B) Post batch combined GEO datasets distribution boxplots. (C) 2D PCA plot of the datasets before debatching. (D) 2D PCA plots of combined GEO datasets after debatching. Green is the disk degeneration dataset GSE34095, and blue is the disk degeneration dataset GSE147383. PCA = principal component analysis.

The research procedure was performed according to the steps outlined in a flowchart (Fig. 1). To obtain the MEMRGs in all samples, the R package sva^[30]^ was used to debatch GSE34095 and GSE147383 to acquire combined GEO datasets, which included 7 IDD samples and 7 controls. To validate the effectiveness of batch effect removal, we normalized the combined GEO datasets and annotated and standardized the probes. First, we compared the differences in expression values before and after-batch effect elimination using a box plot (Fig. 2A and B). Second, the low-dimensional feature distributions of the before-batch and after-batch effect elimination datasets were compared using PCA plots (Fig. 2C and D). The outcomes of the distribution boxplot and PCA plot suggested that batch effects in the IDD dataset were nearly nonexistent after-batch effect elimination.

### 3.2. Determination of MEMRDEGs

**Figure 3.**
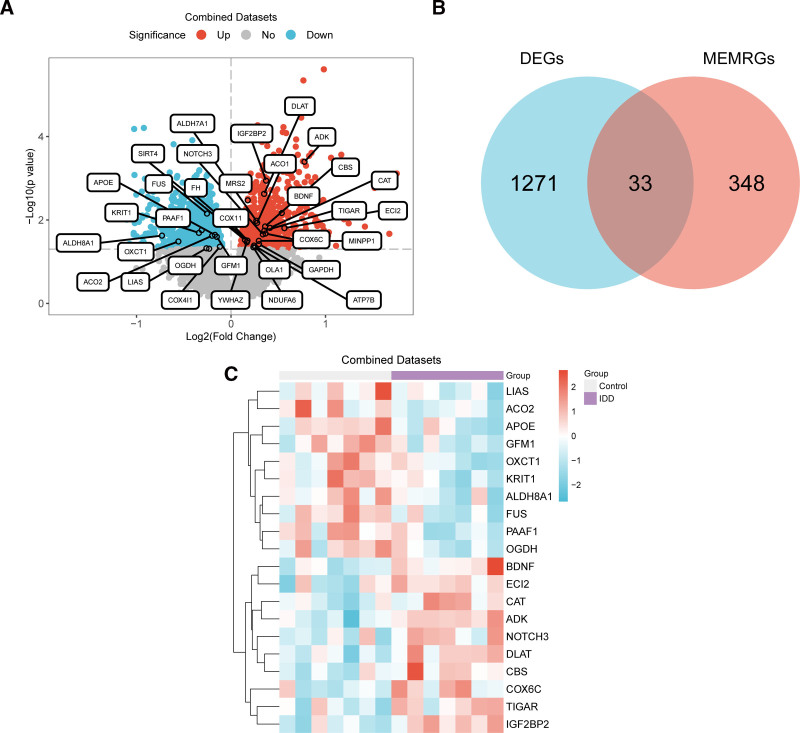
Differential gene expression analysis. (A) Volcano plot of DEGs analysis between the IDD and control groups in the combined GEO datasets. (B) DEGs in the combined GEO datasets, MEMRGs Venn diagram. (C) Heat map of MEMRDEGs in the combined GEO datasets. The purple is the IDD group, and the gray is the control group. In the heat map, red represents high expression, blue represents low expression, and the depth of color represents the degree of expression. DEGs = differentially expressed genes, GEO = Gene Expression Omnibus, IDD = intervertebral disk degeneration, MEMRDEGs = mitochondrial energy metabolism–related differentially expressed genes, MEMRGs = mitochondrial energy metabolism–related genes.

To analyze the differences in gene expression values between the IDD and control groups in the integrated GEO datasets, the R package limma was used for differential analysis of the integrated GEO datasets to obtain DEGs for the 2 groups of data. According to the results, the integrated GEO datasets comprised 1304 DEGs that satisfied the cutoff values of |logFC| > 0 and *P*-value < .05. Within the limits set by this threshold, the expressions of 683 genes were upregulated (logFC > 0 and *P* < .05) and those of 621 genes were downregulated (logFC < 0 and *P* < .05) (Fig. 3A). To detect the MEMRDEGs, we intersected the set of MEMRGs with the set of all DEGs satisfying the criteria of |logFC| > 0 and *P*-value < .05 (Fig. 3B), and then received 33 MEMRDEGs, specific information was shown in Supplementary 2 (Supplemental Digital Content, https://links.lww.com/MD/P866). According to the intersection findings, the expression of MEMRDEGs in the IDD group differed from that in the control group (Fig. 3C).

### 3.3. Differential expression and correlation analysis of MEMRDEGs

**Figure 4.**
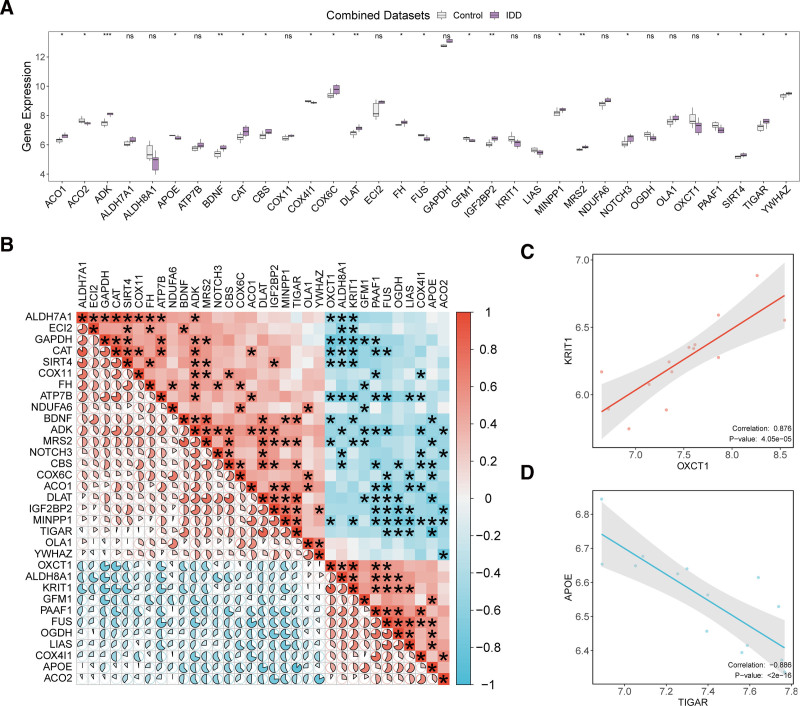
Differential expression and correlation analysis. (A) Group comparison plot of MEMRDEGs in the IDD and control groups. (B) The correlation heat map of MEMRDEGs in the IDD and control groups in the combined GEO datasets. (C) Scatter plot of correlation between MEMRDEGs OXCT1 and KRIT1. (D) Scatter plot of correlation between MEMRDEGs TIGAR and APOE. The absolute value of correlation coefficient (*R* value) below 0.3 was weak or no correlation, 0.3–0.5 was weak correlation, 0.5–0.8 was moderate correlation, and above 0.8 was strong correlation. In the group comparison diagram, purple is the IDD group, and gray is the control group. In the correlation heat map, red is positive correlation, blue is negative correlation, and the depth of color represents the strength of correlation. (ns: nonsignificant, *P < .05, **P < .01, ***P < .001). GEO = Gene Expression Omnibus, IDD = intervertebral disk degeneration, MEMRDEGs = mitochondrial energy metabolism–related differentially expressed genes.

To investigate the disparities in the expression of MEMRDEGs in the IDD and control groups, we compared the expression of 33 MEMRDEGs in the IDD and control groups (Fig. 4A). The differential expression results showed that 21 MEMRDEGs were notably expressed in the IDD and control groups (*P* < .05). These genes included *PDK4*, *CYP4A11*, *ALDH6A1*, *CR1*, *KNG1*, *EHHADH*, *ADH6*, *ACADSB*, *ALDH1L1*, *PC*, *ASS1*, *CKB*, *ECHS1*, *APOE*, *ALDH2*, *AHR*, *UCP2*, *UCHL1*, *HTR2B*, and *ADH1B*. The differential expression results showed that the expression of these genes differed substantially between the IDD and control groups. The expressions of some genes were upregulated in the IDD group, indicating their potential roles in the pathogenesis of IDD, whereas those of others were downregulated, suggesting protective effects against IDD.

We calculated the pairwise correlation of 33 MEMRDEGs in the integrated GEO datasets and found that the MEMRDEGs *OXCT1* and *KRIT1* had a strong direct correlation (*R*-value = 0.876, *P*-value < .001), *TIGAR* and *APOE* had a strong negative correlation (*R*-value = −0.886, *P*-value < .001) (Fig. 4B). Finally, scatter plots of the most strongly positively and negatively correlated gene pairs were generated to visualize the correlation (Fig. 4C and D).

### 3.4. Functional enrichment analysis

**Figure 5.**
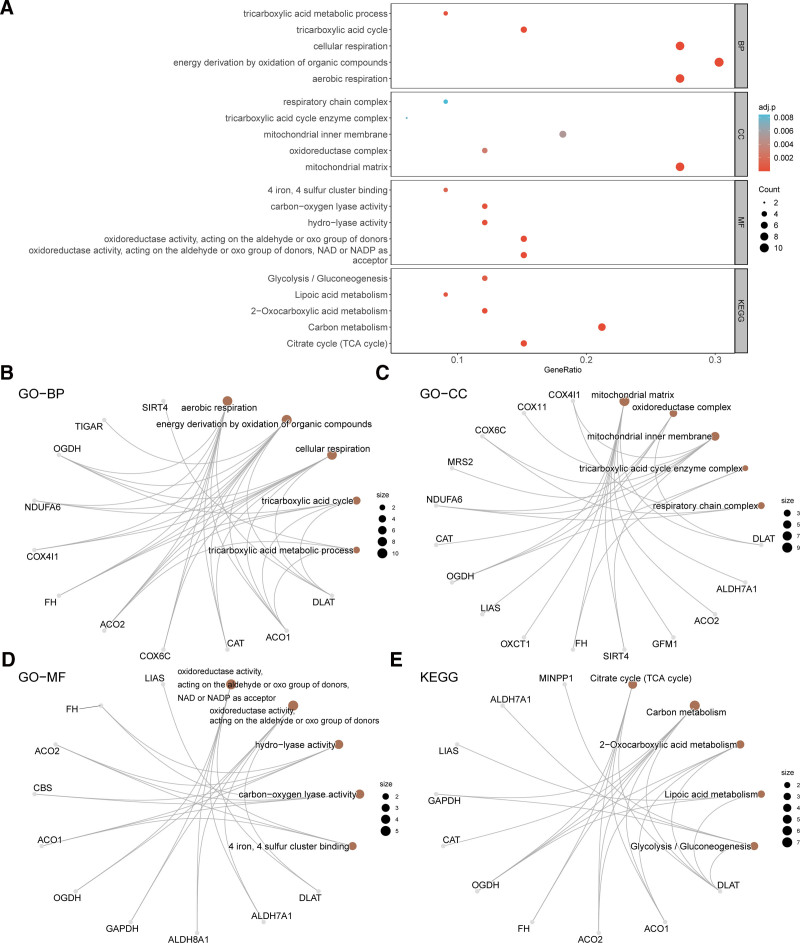
GO and KEGG enrichment analysis for MEMRDEGs. (A) Bubble plot of BP, CC, MF and KEGG. GO terms and KEGG terms are shown on the ordinate. (B–E). GO and KEGG enrichment analysis results of MEMRDEGs. The brown nodes represent items, the gray nodes represent molecules, and the lines represent the relationship between items and molecules. The bubble size in the bubble plot represents the number of genes, and the color of the bubble represents the size of the adj. *P*-value, the redder the color, the smaller the adj. *P*-value, and the bluer the color, the larger the adj. *P*-value. The screening criteria for GO and KEGG enrichment analysis were adj. *P*-value < .05 and FDR value (q-value) < 0.25, and the *P*-value correction method was Benjamini–Hochberg (BH). FDR = false discovery rate, GO = gene ontology, KEGG = Kyoto Encyclopedia of Genes and Genomes, MEMRDEGs = mitochondrial energy metabolism–related differentially expressed genes.

To investigate the BP, CC, MF, and KEGG pathways of the 33 MEMRDEGs in IDD, we first annotated GO and KEGG pathway enrichment analyses of the MEMRDEGs (Table 2). The results demonstrated that 33 MEMRDEGs were primarily enriched in aerobic respiration, energy derivation by oxidation of organic compounds, cellular respiration, the tricarboxylic acid cycle (TCA), tricarboxylic acid metabolic processes, and other BP in IDD. These genes were also enriched in the mitochondrial matrix, oxidoreductase complex, mitochondrial inner membrane, TCA enzyme complex, respiratory chain complex, and other CC, and oxidoreductase activity acting on the aldehyde or oxo group of donors, NAD or NADP as acceptors, oxidoreductase activity acting on the aldehyde or oxo group of donors, hydrolyase activity, carbon-oxygen lyase activity, 4 iron, 4 sulfur cluster binding, and other MF. Additionally, they were enriched in the citrate cycle (TCA cycle), carbon metabolism, 2-OXOCARBOXYLIC acid metabolism, lipoic acid metabolism, glycolysis/gluconeogenesis, and other biological pathways (Fig. 5A–E).

**Table 2:** Result of GO and KEGG enrichment analysis for MEMRDEGs.

Ontology	ID	Description	GeneRatio	BgRatio	*P*-value	*P*.adjust	*q*-value
BP	GO:0009060	aerobic respiration	9/33	196/18,614	4.10E−11	4.05E−08	2.75E−08
BP	GO:0015980	energy derivation by oxidation of organic compounds	10/33	337/18,614	2.12E−10	9.16E−08	6.23E−08
BP	GO:0045333	cellular respiration	9/33	243/18,614	2.79E−10	9.16E−08	6.23E−08
BP	GO:0006099	TCA	5/33	32/18,614	2.48E−09	6.12E−07	4.16E−07
BP	GO:0072350	tricarboxylic acid metabolic process	3/33	14/18,614	1.82E−06	3.60E−04	2.45E−04
CC	GO:0005759	mitochondrial matrix	9/33	484/19,518	7.48E−08	7.10E−06	5.35E−06
CC	GO:1990204	oxidoreductase complex	4/33	128/19,518	6.23E−05	2.96E−03	2.23E−03
CC	GO:0005743	mitochondrial inner membrane	6/33	497/19,518	1.63E−04	5.17E−03	3.89E−03
CC	GO:0045239	TCA enzyme complex	2/33	16/19,518	3.28E−04	7.78E−03	5.87E−03
CC	GO:0098803	respiratory chain complex	3/33	91/19,518	4.83E−04	8.42E−03	6.34E−03
MF	GO:0016620	oxidoreductase activity, acting on the aldehyde or oxo group of donors, NAD or NADP as acceptor	5/33	39/18,369	7.51E−09	1.07E−06	6.96E−07
MF	GO:0016903	oxidoreductase activity, acting on the aldehyde or oxo group of donors	5/33	48/18,369	2.21E−08	1.57E−06	1.02E−06
MF	GO:0016836	hydrolyase activity	4/33	62/18,369	4.47E−06	2.12E−04	1.38E−04
MF	GO:0016835	carbon-oxygen lyase activity	4/33	77/18,369	1.06E−05	3.78E−04	2.47E−04
MF	GO:0051539	4 iron, 4 sulfur cluster binding	3/33	41/18,369	5.38E−05	1.53E−03	9.96E−04
KEGG	hsa00020	Citrate cycle (TCA cycle)	5/28	30/8659	3.27E−08	2.25E−06	1.68E−06
KEGG	hsa01200	Carbon metabolism	7/28	115/8659	5.70E−08	2.25E−06	1.68E−06

BP = biological process, CC = cellular component, GO = gene ontology, KEGG = Kyoto Encyclopedia of Genes and Genomes, MEMRDEGs = mitochondrial energy metabolism–related differentially expressed genes, MF = molecular function, TCA = tricarboxylic acid cycle.

**Figure 6.**
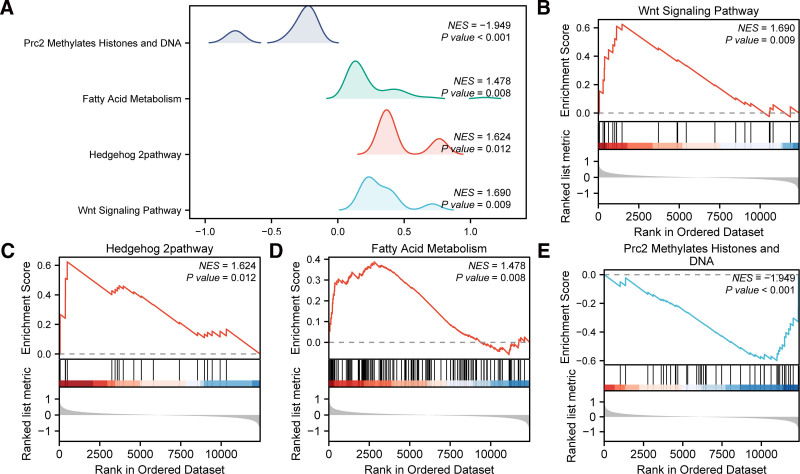
GSEA for combined datasets. (A) GSEA biological functions mountain map presentation of the combined GEO datasets. (B–E) GSEA showed that MEMRDEGs were significantly enriched in the Wnt signaling pathway, Hedgehog 2 pathway, fatty acid metabolism, Prc2 methylates, histones, and DNA. The screening criterion of GSEA was *P*-value < .05. GSEA = gene set enrichment analysis, GEO = Gene Expression Omnibus, MEMRDEGs = mitochondrial energy metabolism–related differentially expressed genes.

To examine the contribution of all gene expression within the integrated GEO datasets to IDD, all genes were subjected to GSEA (Fig. 6A, Table 3). The analysis revealed that all genes present in the integrated GEO datasets exhibited considerable enrichment in the Wnt signaling pathway (Fig. 6B), Hedgehog 2 pathway (Fig. 6C), fatty acid metabolism (Fig. 6D), Prc2 methylates, histones, and DNA (Fig. 6E).

**Table 3:** Results of GSEA for combined datasets.

ID	setSize	EnrichmentScore	NES	*P*-value	*P*.adjust	*q*-value
REACTOME_DNA_STRAND_ELONGATION	31	0.70570	2.08445	1.59E−04	1.58E−02	1.45E−02
REACTOME_RESOLUTION_OF_AP_SITES_VIA_THE_MULTIPLE_NUCLEOTIDE_PATCH_REPLACEMENT_PATHWAY	23	0.75043	2.05797	5.56E−05	7.91E−03	7.28E−03
REACTOME_ELASTIC_FIBRE_FORMATION	38	0.66617	2.05536	3.16E−05	5.87E−03	5.40E−03
BIOCARTA_CELLCYCLE_PATHWAY	23	0.74586	2.04545	8.10E−05	1.09E−02	1.00E−02
WP_RETINOBLASTOMA_GENE_IN_CANCER	84	0.57332	2.03124	6.26E−06	1.68E−03	1.55E−03
PID_PLK1_PATHWAY	43	0.64871	2.02371	3.66E−05	6.14E−03	5.65E−03
REACTOME_POLYMERASE_SWITCHING_ON_THE_C_STRAND_OF_THE_TELOMERE	23	0.73520	2.01621	1.38E−04	1.58E−02	1.45E−02
REACTOME_SYNTHESIS_OF_DNA	108	0.53925	2.01156	1.84E−06	1.11E−03	1.03E−03
REACTOME_MOLECULES_ASSOCIATED_WITH_ELASTIC_FIBRES	33	0.67467	2.01121	2.39E−04	1.81E−02	1.67E−02
REACTOME_RESOLUTION_OF_ABASIC_SITES_AP_SITES	34	0.66597	1.99933	1.49E−04	1.58E−02	1.45E−02
REACTOME_PCNA_DEPENDENT_LONG_PATCH_BASE_EXCISION_REPAIR	20	0.75131	1.99871	2.36E−04	1.81E−02	1.67E−02
NABA_PROTEOGLYCANS	30	0.67972	1.97412	1.53E−04	1.58E−02	1.45E−02
REACTOME_LAGGING_STRAND_SYNTHESIS	20	0.74072	1.97054	3.24E−04	2.01E−02	1.85E−02
REACTOME_POLYMERASE_SWITCHING	14	0.81548	1.96861	1.59E−04	1.58E−02	1.45E−02
PID_TOLL_ENDOGENOUS_PATHWAY	21	0.73309	1.95976	1.80E−04	1.61E−02	1.49E−02
REACTOME_RESOLUTION_OF_SISTER_CHROMATID_COHESION	107	0.52175	1.94139	1.27E−05	3.02E−03	2.78E−03
PID_WNT_SIGNALING_PATHWAY	22	0.62521	1.68969	8.66E−03	1.65E−01	1.52E−01
PID_HEDGEHOG_2PATHWAY	19	0.62341	1.62351	1.23E−02	1.95E−01	1.80E−01
REACTOME_FATTY_ACID_METABOLISM	140	0.38754	1.47836	8.31E−03	1.63E−01	1.50E−01
REACTOME_PRC2_METHYLATES_HISTONES_AND_DNA	45	−0.59862	−1.94892	2.20E−04	1.77E−02	1.63E−02

GSEA = gene set enrichment analysis, NES = normalized enrichment score.

**Figure 7.**
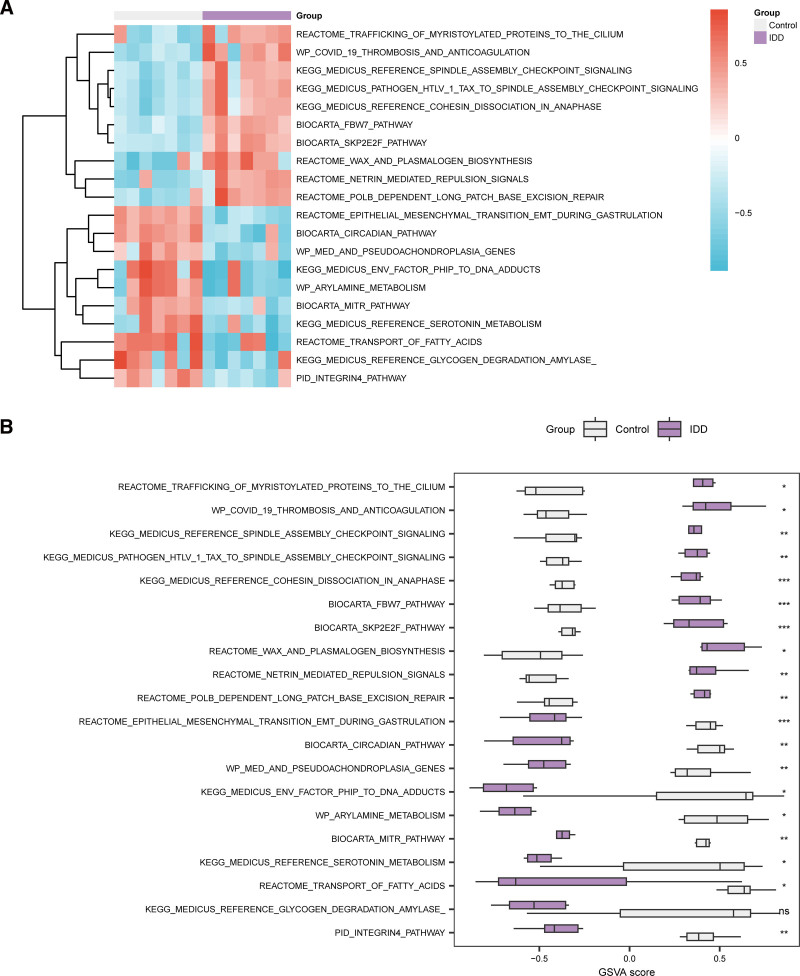
GSVA analysis. (A) Heat map of GSVA results between the IDD and control groups in the combined GEO datasets. (B) Group comparison map of GSVA results between the IDD and control groups in the combined GEO datasets. Purple represents the IDD group and gray represents the control group. Blue represents low enrichment and red represents high enrichment in the heat map. The screening criterion for GSVA was a *P*-value < 0.05. (ns: nonsignificant, *P < .05, **P < .01, ***P < .001). IDD = intervertebral disk degeneration, GSVA = gene set variation analysis, GEO = Gene Expression Omnibus.

We used GSVA to explore the differences in c2.cp.v2023.2. Hs.symbols.gmt gene set between IDD and control groups in the integrated GEO dataset (Table 4). Subsequently, the logFC ranked Top10 positive enriched pathways and Top10 negative enriched pathways with *P*-value < .05 were screened, and the varied expression of 20 pathways between the IDD group and the control group were analyzed and visualized (Fig. 7A). The Mann–Whitney *U* test was performed for differential verification (Fig. 7B). Results showed that the channel integrin pathway, transport of fatty acids, serotonin metabolism, mitr pathway, arylamine metabolism, PhIP to DNA adducts formation, med and pseudoachondroplasia genes, circadian pathway, epithelial–mesenchymal transition during gastrulation, PolB dependent long patch base excision repair, netrin mediated repulsion signals, wax and plasmalogen biosynthesis, Skp2E2F Pathway, Fbw7 Pathway, cohesin dissociation in anaphase, pathogen HTLV-1 tax to spindle assembly checkpoint signaling, spindle assembly checkpoint signaling, COVID-19 thrombosis and anticoagulation, trafficking of myristoylated proteins to the cilium were statistically significant in the IDD group and the control group (*P* < .05).

**Table 4:** Results of GSEA for risk group.

ID	logFC	AveExpr	*t*	*P*-value	adj.*P*.val	*B*
REACTOME_EPITHELIAL_MESENCHYMAL_TRANSITION_EMT_DURING_GASTRULATION	0.8827	−0.0168	6.1825	6.14E−07	2.33E−03	5.6367
KEGG_MEDICUS_ENV_FACTOR_PHIP_TO_DNA_ADDUCTS	0.8807	−0.0719	3.6551	9.03E−04	1.41E−01	−0.6359
BIOCARTA_CIRCADIAN_PATHWAY	0.8499	0.0331	4.9807	2.05E−05	1.94E−02	2.6373
WP_ARYLAMINE_METABOLISM	0.8425	−0.0550	3.8561	5.17E−04	1.11E−01	−0.1546
REACTOME_TRANSPORT_OF_FATTY_ACIDS	0.8068	0.0679	3.3362	2.14E−03	2.20E−01	−1.3800
KEGG_MEDICUS_REFERENCE_SEROTONIN_METABOLISM	0.6914	−0.0675	3.1669	3.35E−03	2.77E−01	−1.7631
KEGG_MEDICUS_REFERENCE_GLYCOGEN_DEGRADATION_AMYLASE_	0.6842	−0.0445	3.0103	5.03E−03	3.26E−01	−2.1091
WP_MED_AND_PSEUDOACHONDROPLASIA_GENES	0.6830	−0.0364	3.8929	4.67E−04	1.11E−01	−0.0657
PID_INTEGRIN4_PATHWAY	0.6540	0.0017	3.9229	4.29E−04	1.09E−01	0.0072
BIOCARTA_MITR_PATHWAY	0.6510	0.0104	3.8112	5.86E−04	1.11E−01	−0.2629
REACTOME_TRAFFICKING_OF_MYRISTOYLATED_PROTEINS_TO_THE_CILIUM	−0.6617	−0.0080	−3.6440	9.31E−04	1.41E−01	−0.6624
REACTOME_POLB_DEPENDENT_LONG_PATCH_BASE_EXCISION_REPAIR	−0.6958	0.0217	−4.0080	3.38E−04	1.07E−01	0.2143
KEGG_MEDICUS_REFERENCE_SPINDLE_ASSEMBLY_CHECKPOINT_SIGNALING	−0.7016	−0.0381	−4.3563	1.25E−04	5.89E−02	1.0726
BIOCARTA_SKP2E2F_PATHWAY	−0.7192	0.0112	−5.0014	1.93E−05	1.94E−02	2.6894
KEGG_MEDICUS_REFERENCE_COHESIN_DISSOCIATION_IN_ANAPHASE	−0.7215	−0.0420	−4.5553	7.06E−05	4.72E−02	1.5690
BIOCARTA_FBW7_PATHWAY	−0.7322	0.0031	−5.1520	1.24E−05	1.94E−02	3.0684
REACTOME_NETRIN_MEDIATED_REPULSION_SIGNALS	−0.7339	−0.0242	−4.2333	1.78E−04	6.76E−02	0.7677
KEGG_MEDICUS_PATHOGEN_HTLV_1_TAX_TO_SPINDLE_ASSEMBLY_CHECKPOINT_SIGNALING	−0.7346	−0.0458	−4.5199	7.82E−05	4.72E−02	1.4804
WP_COVID_19_THROMBOSIS_AND_ANTICOAGULATION	−0.7687	−0.0416	−4.3183	1.40E−04	5.89E−02	0.9783
REACTOME_WAX_AND_PLASMALOGEN_BIOSYNTHESIS	−0.8433	−0.0075	−4.4831	8.70E−05	4.72E−02	1.3885

GSEA = gene set enrichment analysis.

### 3.5. Diagnostic model construction

**Figure 8.**
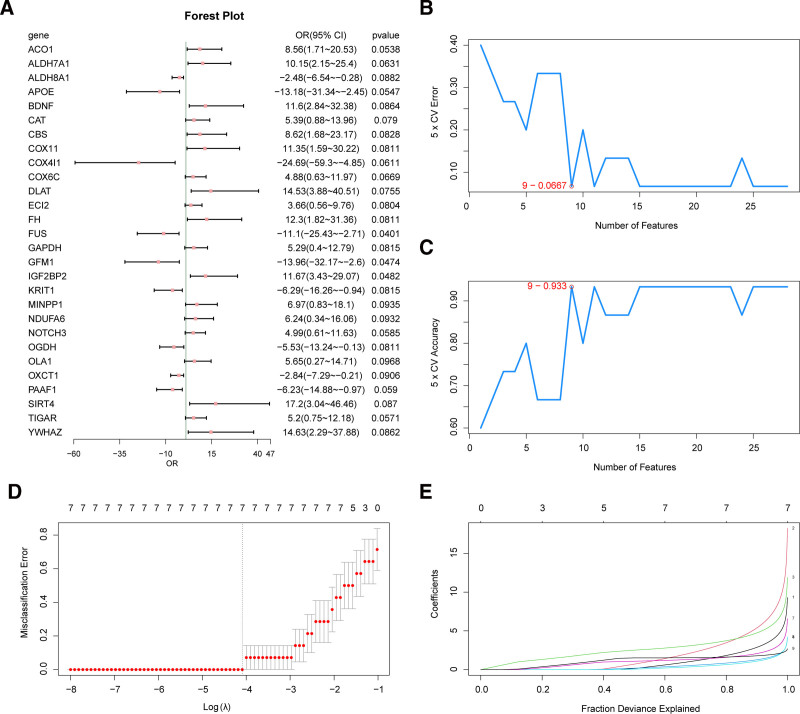
Diagnostic model of IDD. (A) Forest plot of 28 MEMRDEGs included in the Logistic regression model. (B) The number of genes with the lowest error rate obtained by SVM algorithm. (C) The number of genes with the highest accuracy obtained by SVM algorithm. (D) Diagram of variable trajectories of LASSO regression model. (E) Diagnostic model of LASSO regression model. IDD = intervertebral disk degeneration, LASSO = least absolute shrinkage and selection operator, MEMRDEGs = mitochondrial energy metabolism–related differentially expressed genes, SVM = support vector machine.

To determine the diagnostic value of the 33 MEMRDEGs in IDD, we conducted a logistic regression analysis on these genes (Fig. 8A). The results showed that 28 MEMRDEGs were statistically significant in the logistic regression model (*P* < .10). Relying on the 28 MEMRDEGs and the SVM algorithm, we constructed an SVM model to obtain the lowest error rate (Fig. 8B) and highest accuracy rate (Fig. 8C) for the number of genes. The results indicated that when the number of MEMRDEGs was 9, the SVM model had the highest accuracy and lowest error rate for *NDUFA6*, *YWHAZ*, *DLAT*, *BDNF*, *ECI2*, *COX4I1*, *ACO1*, *COX11*, and *ALDH7A1*. Next, based on the 9 MEMRDEGs included in the SVM model, LASSO regression analysis was performed to establish the LASSO regression model (Fig. 8D) and LASSO coefficient profile (Fig. 8E). The LASSO regression model, which served as a diagnostic model for IDD, showed that the following 7 MEMRDEGs were key genes: *NDUFA6*, *YWHAZ*, *DLAT*, *BDNF*, *ECI2*, *ACO1*, and *ALDH7A1*.

### 3.6. Validation of diagnostic model

**Figure 9.**
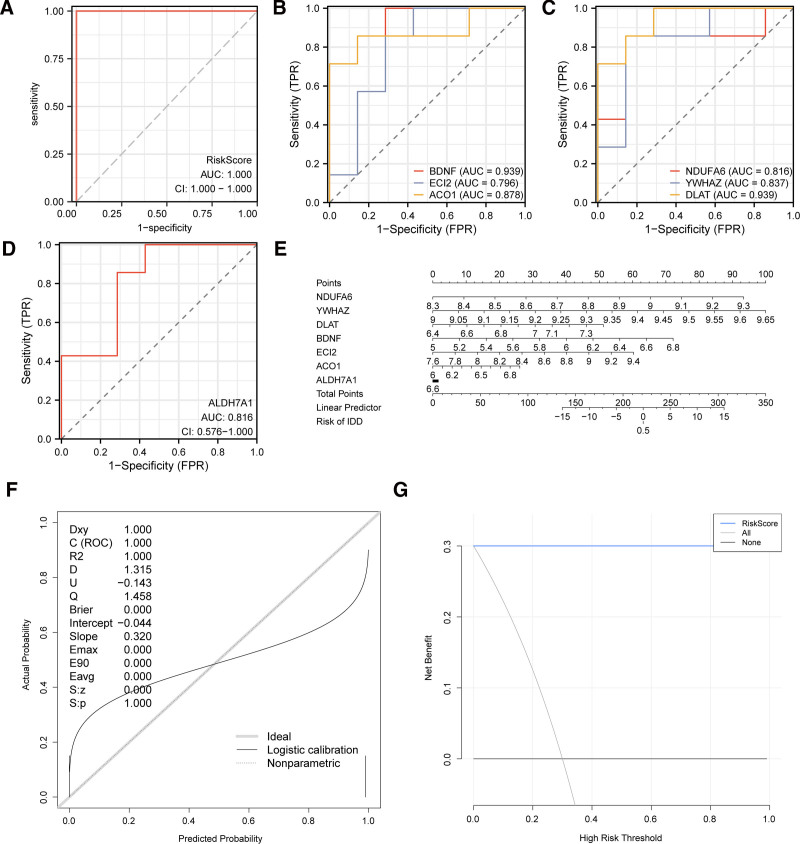
ROC curve and model validation analysis of IDD. (A) ROC curve of risk score in combined GEO datasets. (B–D) ROC curves of key genes in the IDD and control groups. (E) Nomograms of key genes in combined GEO datasets for IDD diagnostic model. (F) Calibration curve plot of key genes in combined GEO datasets for IDD diagnostic model. (G) DCA plot of of key genes in combined GEO datasets for IDD diagnostic model. The ordinate of the calibration curve plot is the net benefit, and the abscissa is the probability threshold or threshold probability. When AUC was above 0.9, it had a high accuracy, and when AUC was 0.7–0.9, it had a certain accuracy. AUC = area under the curve, DCA = decision curve analysis, GEO = Gene Expression Omnibus, IDD = intervertebral disk degeneration, ROC = receiver operating characteristic.


To further corroborate the value of the IDD diagnostic model, we generated a ROC curve using the risk scores derived from the amalgamated GEO datasets. The ROC curve showed (Fig. 9A) that the expression of the risk score in the integrated GEO datasets had high accuracy (AUC > 0.9) in differentiating between different groups. The formula for calculating the risk score is as follows:

$$\begin{array}{l} \begin{array}{llll} & Risk\;score=ACO1*\left(2.7274\right)+ALDH7A1*\left(1.9738\right)+BDNF*\left(1.7344\right)\; & +DLAT*\left(5.9422\right)+ECI2*\left(1.6084\right)+NDUFA6*\left(4.0055\right)\; & +YWHAZ*\left(7.5364\right)\; \end{array} \end{array}$$

After dividing the IDD group into high- and low-risk groups based on the median risk score, we used the number of key genes to plot the ROC curve and evaluate their diagnostic accuracy. The results (Fig. 9B–D) indicated that *BDNF* and *DLAT* had higher accuracy, whereas *ACO1*, *ALDH7A1*, *ECI2*, *NDUFA6*, and *YWHAZ* also exhibited certain accuracy, which may not be as accurate as *BDNF* and *DLAT*.

Based on these key genes, we constructed a nomogram to show the relationships between the key genes in the integrated GEO datasets (Fig. 9E). The findings revealed that the expression of the crucial gene *YWHAZ* exhibited notably greater usefulness in the diagnostic model for IDD compared with that of other variables, whereas the expression of the key gene, *ALDH7A1*, showed significantly lower utility for the diagnosis model of IDD compared to other variables. Thereafter, we used calibration analysis to evaluate the accuracy and discrimination of the diagnosis model of IDD (Fig. 9F), the calibration line represented by the dashed line, aligns well with the diagonal line of the ideal model, suggesting that the IDD diagnosis model possesses predictive accuracy for real-world outcomes. We also used DCA to assess the clinical usefulness of the diagnostic model for detecting IDD (Fig. 9G). The data indicated that, within a certain range, the model’s line was steadily superior to all positive and negative lines, and the model had more net benefits, indicating good performance.

### 3.7. Friends analysis

**Figure 10.**
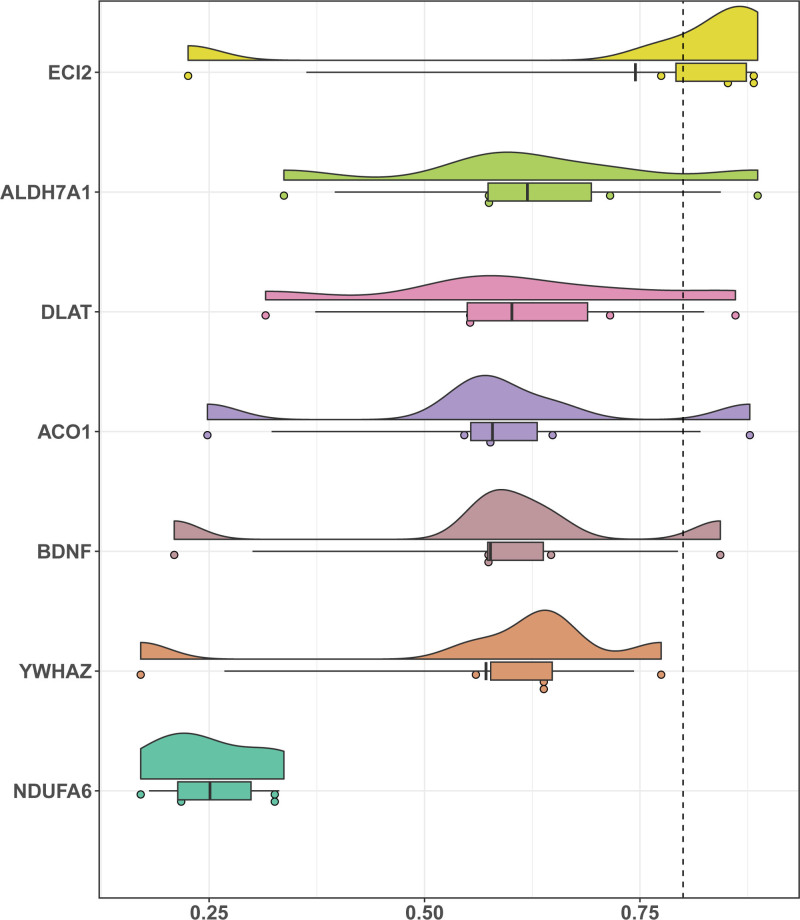
Friends analysis of key genes. Cloud and rain diagram of friends analysis results of key genes.

Functional similarity (friends) was used to evaluate the importance of genes in the BPs of IDD (Fig. 10). The results indicated that *ECI2* plays an important role in IDD and is the gene closest to the cutoff value (cutoff value = 0.80).

### 3.8. Regulatory network construction

**Figure 11.**
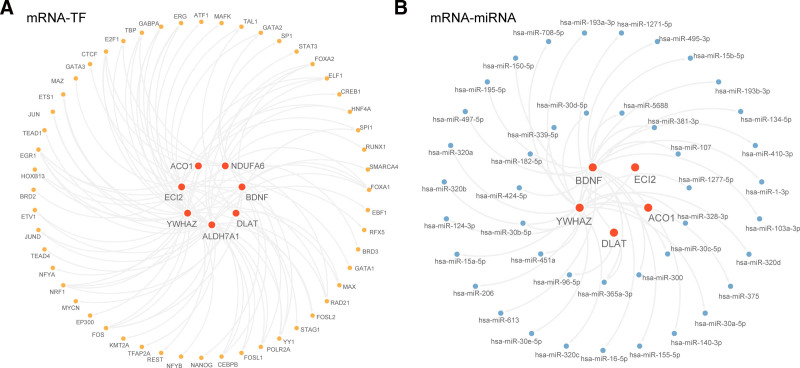
Regulatory network analysis of key genes. (A) mRNA–TF regulatory network of key genes. (B) mRNA–miRNA regulatory network of key genes. Key genes are in red, TFs are in orange, and miRNAs are in blue. TFs = transcription factors.

Using the ChIPBase database, we identified TFs associated with key genes. The screening criteria were as follows: number of samples found (upstream and downstream) > 5. We constructed an mRNA–TF regulatory network (Fig. 11A) which included 7 key genes and 52 TFs (Supplementary 3, Supplemental Digital Content, https://links.lww.com/MD/P866). Subsequently, we retrieved the miRNAs related to key genes from the StarBase database. The screening criteria were set as having at least 4 source records for mRNA–miRNA interaction relationships to construct the mRNA–miRNA regulatory network (Fig. 11B), which contained 5 key genes and 42 miRNAs (Supplementary 4, Supplemental Digital Content, https://links.lww.com/MD/P866).

### 3.9. Analysis of immune infiltration

**Figure 12.**
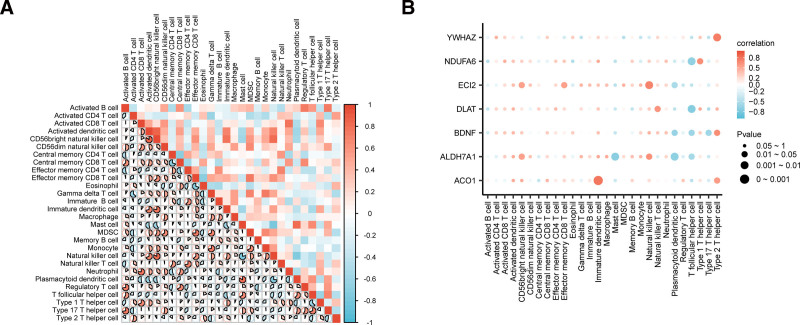
Immune infiltration analysis by ssGSEA algorithm. (A) Correlation heatmap of immune cell infiltration abundance in the combined GEO datasets. (B) Bubble plot of correlation between key genes and immune cell infiltration abundance in the combined GEO datasets. The absolute value of correlation coefficient (*R*-value) below 0.3 was weak or no correlation, 0.3–0.5 was weak correlation, 0.5–0.8 was moderate correlation, and above 0.8 was strong correlation. Red is positive correlation, blue is negative correlation. The depth of the color represents the strength of the correlation. GEO = Gene Expression Omnibus, ssGSEA = single-sample gene set enrichment analysis.

First, a heatmap of correlation was used to showcase the outcomes of the immune infiltration analysis among the 28 immune cell types in the integrated GEO datasets (Fig. 12A). The findings showed that most immune cells exhibited strong correlations, and immune cells stimulated by dendritic cells and CD56bright natural killer cells had the strongest positive correlation (*R*-value = 0.842, *P*-value < .05).

A bubble plot illustrating the correlation revealed the relationship between the expression of vital genes and the quantities of immune infiltration. (Fig. 12B). The correlation bubble plot showed that most immune cells exhibited strong correlations, and *ACO1* and immune cell immature dendritic cells had the strongest positive correlation (*R* = 0.780, *P* < .05).

## 4. Discussion

IDD is a frequently occurring degenerative condition and a primary contributor to LBP, which tends to worsen with age. Among people aged 50 years and older, over 80% exhibit changes associated with IDD.^[31]^ These changes might not only lead to chronic pain but could also restrict patients’ mobility, affect their work and daily life, and impose a considerable economic burden on society.^[32]^ The currently available treatment strategies for IDD primarily include physical therapy, pharmacological therapy, and surgery, which have achieved some success in symptom control but have limited effects in reversing IDD progression. Therefore, future studies should delve deeper into the pathogenesis of IDD and seek more effective prevention and treatment strategies. The key BPs related to this condition include innate immune responses, cell division, mitochondrial homeostasis, and cell proliferation. Abnormal mitochondrial energy metabolism and immune cell infiltration play crucial roles in accelerating IDD. However, comprehensive research on abnormal mitochondrial energy metabolism in IDD is lacking.

In this study, we explored the potential molecular mechanisms of intervertebral disc degeneration (IDD) based on publicly available GEO datasets (GSE34095 and GSE147383) and constructed a corresponding diagnostic model. Due to the scarcity of available datasets related to IDD, we selected these 2 datasets after a rigorous literature search and screening, and considered them to have high data quality and representativeness. Despite the limited sample size, we minimized batch effects and errors through reasonable data integration and analysis strategies to ensure the reliability of the study conclusions. In data processing, we used the GEOquery package to collect data and combined it with the limma package for standardization, and PCA to correct for batch effects in order to improve the comparability of different datasets. In the model evaluation, we verified the diagnostic value of key genes by ROC curve analysis of genes (AUC > 0.7 in all cases), and further evaluated the stability and clinical applicability of the model by DCA and calibration curve, which showed that the model had a better diagnostic performance on the existing dataset.

We attempted to identify diagnostic markers of IDD and investigate the effect of abnormal mitochondrial energy metabolism and immune cell infiltration on these conditions. Mitochondrial energy metabolism serves as the foundation for cellular activities and is crucial for maintaining normal cellular functions and health. Therefore, abnormalities in mitochondrial energy metabolism may be closely associated with various congenital diseases, metabolic disorders, inflammation, and tumors.^[33,34]^ Enhanced mitochondrial energy metabolism leads to increased production of reactive oxygen species (ROS) during energy-generating processes. High levels of ROS can be harmful and damage intracellular molecules and structures. Conversely, a decline in mitochondrial energy metabolism can affect multiple organ function, reduce immunity, and contribute to metabolic disorders, neurological and muscular issues, obesity, and impairment of cell division and DNA synthesis. Interestingly, abnormal mitochondrial energy metabolism may be pivotal in IDD progression.^[35]^ Research has shown that immune cell infiltration can influence IDD progression.^[11]^ Over the past few decades, researchers have made considerable efforts to elucidate the relationship between IDD and immune cells.^[36]^ Protrusion of the NP of the intervertebral disk may affect autoimmune responses via its association with T cells, B cells, and neutrophils.^[37]^ Further studies are required to understand the complexity of the immune microenvironment in patients with IDD. Our findings reveal that IDD progression is associated with the infiltration of various immune cells, including mast cells, CD56bright natural killer cells, immature dendritic cells, plasmacytoid dendritic cells, and follicular helper T cells. In addition, we constructed the mRNA–miRNA regulatory network to explore its role in the regulation of mitochondrial function and the progression of IDD. It has been shown that specific miRNAs play important regulatory roles in mitochondrial energy metabolism and inflammatory pathways; therefore, the construction of this network can help to reveal the underlying molecular mechanisms and provide candidate targets for future studies. Although this study was limited by data scarcity, we will further collect larger clinical samples in the future to validate the robustness of the model and expand the depth of analysis in combination with high-throughput sequencing data (RNA-seq) to further reveal the key molecular mechanisms of IDD.

Using comprehensive bioinformatic analysis, we identified 7 genes related to mitochondrial energy metabolism and successfully constructed a model that effectively predicted IDD. These genes include *NDUFA6*, *YWHAZ*, *DLAT*, *BDNF*, *ECI2*, *ACO1*, and *ALDH7A1*. These key genes hold significant clinical value for the early diagnosis and subsequent progression of IDD. Among them, *BDNF*, *DLAT*, and *YWHAZ* exhibit high diagnostic value for the early detection of IDD, whereas *ECI2* is a key factor in the deterioration of the disease. BDNF is a protein that plays a vital role in the central nervous system. It promotes the growth, differentiation, and survival of neurons, particularly in memory, learning, and higher cognitive functions. Studies have shown^[38]^ that chronic stress can lead to a reduction in *BDNF* signaling in the limbic regions of the cerebral cortex. This decrease in signal transduction can diminish mitochondrial energy metabolism and play a significant role in the early stages of degenerative diseases. *BDNF* has been implicated in various disorders, including neurodegenerative diseases^[39,40]^ and cardiac hypertrophy.^[41]^ Under these conditions, *BDNF* expression is reduced, potentially via 2 mechanisms: first, increased methylation of the *BDNF* promoter leads to transcriptional repression, and second, specific microRNAs (such as miR-212/132) bind more frequently to *BDNF* mRNA, resulting in the downregulation of its expression. Consequently, *BDNF* has emerged as a potential therapeutic target for treating these diseases. Although there seems to be no direct association between *BDNF* and IDD, considering the potential nerve compression and inflammatory responses that might accompany the process of IDD, as well as the negative effect of long-term chronic pain caused by IDD on the central nervous system, we speculated that *BDNF* might indirectly participate in this process. *DLAT* is a subunit of the pyruvate dehydrogenase complex (PDC), primarily located within the mitochondria and catalyzes the irreversible oxidative decarboxylation of pyruvate to acetyl-CoA. This enzyme plays a crucial role in mitochondrial energy metabolism and causes mutations in human metabolic syndromes.^[42]^ *DLAT* also regulates the activation of pyruvate dehydrogenase complex via its interaction with p32 in the mitochondria. In TCGA cohort, a noteworthy association exists between low expression of p32 and *DLAT*, overall survival, and disease-specific survival outcomes of patients.^[43]^ The oxidation process involving *DLAT* has important implications for the intracellular redox balance and ROS generation. Abnormal ROS levels are closely associated with the onset and progression of various degenerative diseases. Therefore, *DLAT* may be linked to the progression of degenerative diseases via these mechanisms.^[44]^ As a common degenerative disease, IDD is likely mediated by *DLAT* in its occurrence and development, making it a potential target for future research. Further validation is needed to confirm the precise role of *DLAT* in mitochondrial energy metabolism, oxidation reactions, and other processes as well as to explore its clinical application as a biomarker. In this study, the ROC curve and AUC values were used to verify the effectiveness of the IDD diagnostic model, confirming that the *BDNF* and *DLAT* genes are highly reliable in distinguishing between high- and low-risk groups of patients with IDD. This study provides valuable references for early diagnosis, disease monitoring, and treatment strategies for IDD. *YWHAZ* is an abundant signaling protein responsible for receiving, transmitting, and regulating intracellular signals, and participates in many important cellular signaling pathways. Changes in *YWHAZ* expression or function may disrupt normal cellular homeostasis, leading to age-related decline or neurodegenerative diseases such as AD and PD.^[45]^ Although the importance of *YWHAZ* in various cellular metabolic processes and diseases has been widely recognized, its specific role in IDD requires further research and elucidation. Understanding how *YWHAZ* contributes to these processes may lead to the development of innovative treatment strategies for IDD and other age-related diseases. We comprehensively evaluated the performance of the IDD diagnostic model based on key genes by constructing a nomogram, calibration curve, and decision curve. These results indicate that the model has high accuracy and clinical practicality in predicting IDD, particularly the expression of the *YWHAZ* gene, which may play an important role in diagnosis. These findings provide valuable information for the initial diagnosis and personalized treatment of IDD. *ECI2* is a cofactor for the oxidation of unsaturated fatty acids. Studies have reported that upregulation of *ECI2* expression can promote the progression of prostate cancer, and its high expression is considered a potential therapeutic target for prostate cancer.^[46]^ However, the specific role and mechanism of action of *ECI2* in IDD remain unclear and require further investigation. We conducted a functional similarity analysis to assess the key genes that play significant roles in the BPs of IDD. These results indicate that *ECI2* is closely associated with mitochondrial energy metabolism and plays a crucial role in the progression of IDD, potentially becoming an important target for future therapeutic and diagnostic strategies. Although *NDUFA6*, *YWHAZ*, *DLAT*, *BDNF*, *ECI2*, *ACO1*, and *ALDH7A1* have not been extensively studied in IDD, further validation may reveal their potential as novel therapeutic targets.

The NP tissue of the intervertebral disk is located under the protection of the AF and CEP, forming a relatively closed environment. This special structural layout isolates nuclear pulposus cells and the secreted ECM from immune cells under normal circumstances. However, when IDD occurs, the integrity of the NP is compromised and its internal contents may leak into the periphery. When these NP effluents come in contact with the external immune system, they are mistakenly recognized as foreign antigens that trigger an initial immune response. Gradually, if the NP tissue continues to be damaged, granulation tissue may form during the repair process. The formation of this granulation tissue allows external blood vessels to grow into the damaged area, leading to the direct exposure of the NP tissue to circulating immune cells. In this situation, ECM components secreted by the NP cells, such as collagen and proteoglycans, are recognized as self-antigens by the immune system and trigger secondary immune responses. Neutrophils, T cells, macrophages, and regulatory T cells (Tregs) are immune cells that play vital roles in mediating the immune reaction of the intervertebral disk. When these cells infiltrate the intervertebral disk tissue, they release a large number of pro-inflammatory chemokines, triggering a series of inflammatory reactions. This inflammatory cascade not only intensifies the degree of inflammation within the intervertebral disk but also accelerates structural degradation of the disk, ultimately leading to disk dysfunction.^[47,48]^ Using ssGSEA, we assessed the degree of infiltration by immune cells and found that a range of immune cells were involved in the progression of IDD, which shows a strong correlation with 7 key genes. This discovery revealed an intricate interaction between IDD and the immune system and confirmed the involvement of MEMRDEGs in the degeneration process. This deepens our understanding of the pathogenesis of IDD. Therefore, identifying the molecules related to the inflammatory environment and immune cell infiltration in degenerated intervertebral disks is crucial for revealing the underlying mechanisms of IDD pathogenesis.

In this study, 7 key genes (NDUFA6, YWHAZ, DLAT, BDNF, ECI2, ACO1 and ALDH7A1) were screened by integrating bioinformatics analysis and machine learning methods, and a diagnostic model was constructed based on these genes. The validation results of the ROC curves and AUC values of the model showed that the gene combination had high accuracy in distinguishing high-risk and low-risk IDD patients, suggesting its clinical application value as a potential biomarker. In practical clinical applications, this gene model can be used for the detection of blood or tissue samples and combined with imaging tests (e.g., MRI, X-ray) to improve the early diagnosis rate of IDD and the accuracy of the prediction of disease progression, so as to optimize the personalized treatment strategies for patients. In addition, our study revealed that the progression of IDD is closely related to immune cell infiltration, especially the involvement of immune cells such as mast cells, CD56bright natural killer cells and dendritic cells. These findings suggest that an abnormal immune microenvironment may play a key role in the pathologic process of IDD. Future therapeutic strategies may focus on the modulation of the immune microenvironment, such as targeted inhibition of pro-inflammatory immune factors, to reduce disc damage caused by inflammatory responses. In addition, immune checkpoint inhibitors or blockers of specific inflammatory pathways may become a new direction for IDD treatment. To further validate the reliability of the proposed IDD diagnostic model, we plan to collect larger clinical samples in subsequent studies to assess the stability and clinical feasibility of the model. Meanwhile, we will further explore the specific regulatory mechanisms of key genes (e.g., BDNF, DLAT, YWHAZ, and ECI2) in the progression of IDD and evaluate the feasibility of these genes as potential therapeutic targets in combination with drug screening techniques.

To further explore the diagnostic markers for IDD, we employed various statistical and machine learning methods, including logistic regression, SVM algorithms, and LASSO logistic regression. Additionally, we used ssGSEA to investigate the infiltration of immune cells in IDD. However, our study has several limitations. First, we relied entirely on publicly available datasets that often have relatively small sample sizes. This could have potentially led to higher false-positive rates and biases in our research findings. Second, our study did not consider important factors such as gender, ethnicity, and age. Therefore, in future studies, we plan to integrate multiple independent databases to enhance the statistical power of our research and ensure the accuracy of our findings using large-scale experimental validation. Simultaneously, we will endeavor to collect more clinical samples to validate the practical application of the diagnostic models we have constructed. In addition, we are aware of the importance of performing multiple hypothesis testing corrections and will explore more rigorous screening such as false discovery rate correction (p-adj) in subsequent studies to ensure the robustness and reproducibility of the findings. By addressing these limitations, we aimed to improve the reliability and generalizability of our research on IDD diagnostic markers.

## 5. Conclusion

To summarize, we identified 7 key genes (*NDUFA6*, *YWHAZ*, *DLAT*, *BDNF*, *ECI2*, *ACO1*, and *ALDH7A1*) that are associated with IDD. Furthermore, the findings of this study imply that the infiltration of immune cells may have a potential impact on the pathogenesis and progression of IDD. By regulating mitochondrion-related gene expression to interfere with mitochondrial energy metabolism, new potential therapeutic targets for IDD treatment can be explored.

## Acknowledgments

We are grateful to the dedication and hard work of all participants during the data mining phase.

## Author contributions

**Methodology:** Zhenwei Wang.

**Writing – original draft:** Jianlan Lv.

## Supplementary Material


