# Supplementary material for: Comprehensive analysis of mitochondrial energy metabolism–related genes and immune infiltration in intervertebral disk degeneration
Source: Medicine (Baltimore). 2025 Sep 5;104(36):e44306. doi: 10.1097/MD.0000000000044306 (PMC12419262; doi:10.1097/MD.0000000000044306)
Supplement: Supplementary file 1 [file medi-104-e44306-s001.docx]

***Supplementary 1*. The 381 MEMRGs data.**

| x |
| --- |
| C1QBP |
| TIGAR |
| GFM2 |
| HTT |
| CS |
| TANGO2 |
| LIAS |
| COX5A |
| NFATC4 |
| ETFA |
| PPARGC1A |
| AK2 |
| CYCS |
| SLC25A20 |
| SDHB |
| OGDH |
| SLC25A3 |
| ETFB |
| FBXL4 |
| PTEN |
| PDP1 |
| UCP2 |
| COQ9 |
| TP53 |
| SOD1 |
| TLR2 |
| CNR1 |
| SCO2 |
| AK3 |
| UCP1 |
| UCP3 |
| TCF19 |
| DNAJC19 |
| LDHA |
| EIF4E |
| RPS6KB1 |
| KNG1 |
| PRKCZ |
| POLG |
| DLAT |
| EIF4EBP1 |
| ACAD9 |
| CR1 |
| KLK4 |
| TNNT1 |
| CD200 |
| OMA1 |
| SUCLA2 |
| TUFM |
| GFM1 |
| AUH |
| HIBCH |
| TSFM |
| TAFAZZIN |
| TMEM70 |
| MTIF2 |
| OPA3 |
| SERAC1 |
| CENPO |
| MTIF3 |
| LIX1 |
| C2orf88 |
| NDUFS4 |
| NDUFV1 |
| NFU1 |
| LIPT2 |
| FH |
| LIPT1 |
| IBA57 |
| SRC |
| SIRT3 |
| ESRRA |
| TPK1 |
| AGK |
| MFF |
| AIFM1 |
| BDNF |
| NR1H4 |
| CRAT |
| ECHS1 |
| NDUFS1 |
| NGLY1 |
| DHTKD1 |
| GPBAR1 |
| CHCHD10 |
| PKHD1 |
| UCHL1 |
| GSK3B |
| ASS1 |
| MFN2 |
| PRKN |
| ATP7B |
| IRS1 |
| PAH |
| SCN9A |
| SOD2 |
| TPI1 |
| KCNK9 |
| ACADVL |
| GCDH |
| VDAC1 |
| ATP5F1B |
| BTD |
| OCRL |
| NDUFS2 |
| RARS2 |
| KRIT1 |
| MIPEP |
| SUMF1 |
| FLVCR2 |
| SLC27A1 |
| BOLA3 |
| SZT2 |
| PPARGC1B |
| MITD1 |
| FAM210B |
| ESR1 |
| CAT |
| FOXO1 |
| PSEN1 |
| ATP2A2 |
| CBS |
| NOTCH3 |
| ADK |
| APOE |
| EIF2AK3 |
| GLS |
| PGK1 |
| STIM1 |
| YWHAE |
| CASP7 |
| DLD |
| HPRT1 |
| HSPD1 |
| KCNJ2 |
| PKD2 |
| RYR1 |
| SDHA |
| SLC25A4 |
| ADORA2B |
| HSPA9 |
| HTR2A |
| IL1B |
| INS |
| KCNJ5 |
| RYR2 |
| VCL |
| YWHAZ |
| ACOX1 |
| ADORA2A |
| COX4I1 |
| FXN |
| HK2 |
| HUWE1 |
| NDUFS3 |
| NEU1 |
| PRDX6 |
| HTR3A |
| IDH3A |
| PDHX |
| UQCRC2 |
| XBP1 |
| BSG |
| CKB |
| EIF2S1 |
| FUS |
| IGF2BP2 |
| SHMT1 |
| SIRT5 |
| ATG7 |
| BAD |
| CLOCK |
| DBT |
| ESRRG |
| GDAP1 |
| ID2 |
| PNPLA6 |
| SLC25A5 |
| TFAM |
| UQCRC1 |
| CPT1C |
| EPO |
| HTR2B |
| LRPPRC |
| NME4 |
| PDK4 |
| PROK2 |
| PTPA |
| SOAT2 |
| BICD2 |
| DMRT1 |
| INF2 |
| NDUFAF1 |
| OLA1 |
| RYR3 |
| SSBP1 |
| SURF1 |
| GLRX5 |
| IMMT |
| MAVS |
| NDUFA5 |
| NDUFS5 |
| SIRT4 |
| SMPD2 |
| ATP5PF |
| NDUFAF3 |
| ACAD10 |
| ALYREF |
| COX7A2L |
| MRS2 |
| TIMMDC1 |
| ATP5MC1 |
| FAHD1 |
| SLC25A27 |
| OCIAD1 |
| TUG1 |
| ACADL |
| ALDH18A1 |
| ADH1B |
| PPAT |
| GAPDH |
| ALDH2 |
| ACSL4 |
| PPARG |
| CYP2U1 |
| PFKP |
| ACSL1 |
| ADH1A |
| GPI |
| ALDH3B2 |
| NDUFS6 |
| CYP4A22-AS1 |
| PC |
| MDH2 |
| CYC1 |
| PFKFB2 |
| ACAT1 |
| CPT1B |
| ACAA2 |
| CYP4A26P |
| NDUFB4 |
| ACAA1 |
| MDH1B |
| NDUFS8 |
| NDUFAB1 |
| ALDH3B1 |
| ATP4A |
| COX5B |
| PFKP-DT |
| ACSBG1 |
| COX6C |
| NDUFB9 |
| ALDH1B1 |
| ALDH1A2 |
| NDUFA9 |
| CYP4A11 |
| NDUFB11 |
| NDUFA12 |
| PFKFB4 |
| IDH1 |
| ACADSB |
| ATP4B |
| ALDH3A1 |
| PAAF1 |
| ACADS |
| PPAN |
| SDHC |
| MDH1 |
| PGM2 |
| PFKFB3 |
| NDUFB5 |
| ALDH1L2 |
| PKLR |
| NDUFA8 |
| HADHA |
| NDUFB3 |
| ALDH1L1 |
| CPT1A |
| CYP4A22 |
| CYP4A27P |
| ADH1C |
| EHHADH |
| NDUFB10 |
| OXCT2 |
| OXCT1 |
| ACADM |
| ALDH4A1 |
| PDHB |
| NDUFA7 |
| COX7B |
| UQCRFS1 |
| ACLY |
| PFKL |
| IDH3G |
| ACOX2 |
| NDUFA10 |
| PFKFB1 |
| GAPDHS |
| ECI1 |
| COX10 |
| ALDH8A1 |
| ALDH6A1 |
| NDUFA11 |
| CYP4A44P |
| ALDH9A1 |
| ADH4 |
| NDUFA6 |
| MINPP1 |
| AKR1A1 |
| ACO1 |
| NDUFV2 |
| NDUFA13 |
| NDUFA1 |
| PDC |
| ADPGK |
| IDH3B |
| PPATP1 |
| ATP12A |
| ECI2 |
| CPT2 |
| AHR |
| MDH1P2 |
| CYP4A43P |
| HMGCL |
| POR |
| GCK |
| DLST |
| COX17 |
| HADHB |
| ACOX3 |
| ALDH1L1-AS1 |
| ALDH3A2 |
| LHPP |
| PPARA |
| ACSL6 |
| PPARD |
| ACSBG2 |
| NDUFB7 |
| ALDH5A1 |
| HADH |
| NDUFS7 |
| ACSL6-AS1 |
| ALDH1A1 |
| PPA2 |
| NDUFA4 |
| COX15 |
| PFKM |
| NDUFC2 |
| BPGM |
| ADH6 |
| NDUFV3 |
| ACSL3 |
| ACAT2 |
| ALDH16A1 |
| SDHD |
| NDUFB2 |
| ACSL3-AS1 |
| NDUFB6 |
| NDUFC1 |
| ALDH7A1P2 |
| COX7C |
| GALM |
| ALDH7A1P4 |
| PPATP2 |
| ALDH7A1 |
| ALDH1A3 |
| ACSL5 |
| PPAN-P2RY11 |
| ALDH7A1P3 |
| NDUFB8 |
| ALDH1L1-AS2 |
| ALDH7A1P1 |
| ACO2 |
| OXCT1-AS1 |
| COX11 |
| OXCT2P1 |
| NDUFA2 |
| NDUFA3 |
| NDUFB1 |
| PPA1 |
| MDH1P1 |

***Supplementary 2*. The 33 MEMRDEGs data.**

| Gene |
| --- |
| ADK |
| IGF2BP2 |
| DLAT |
| MRS2 |
| BDNF |
| FUS |
| ALDH7A1 |
| ACO1 |
| CBS |
| CAT |
| ECI2 |
| PAAF1 |
| TIGAR |
| KRIT1 |
| COX6C |
| NOTCH3 |
| APOE |
| ALDH8A1 |
| ACO2 |
| GFM1 |
| SIRT4 |
| COX11 |
| FH |
| MINPP1 |
| OXCT1 |
| YWHAZ |
| GAPDH |
| ATP7B |
| COX4I1 |
| NDUFA6 |
| OLA1 |
| LIAS |
| OGDH |

***Supplementary 3*. The data of seven key genes and 52 TFs .**

| mRNA | TF |
| --- | --- |
| ACO1 | CTCF |
| ACO1 | EGR1 |
| ACO1 | NRF1 |
| ALDH7A1 | CEBPB |
| ALDH7A1 | CTCF |
| ALDH7A1 | EBF1 |
| ALDH7A1 | EGR1 |
| ALDH7A1 | ELF1 |
| ALDH7A1 | ERG |
| ALDH7A1 | ETS1 |
| ALDH7A1 | ETV1 |
| ALDH7A1 | FOS |
| ALDH7A1 | FOSL1 |
| ALDH7A1 | FOSL2 |
| ALDH7A1 | FOXA1 |
| ALDH7A1 | FOXA2 |
| ALDH7A1 | GABPA |
| ALDH7A1 | JUN |
| ALDH7A1 | JUND |
| ALDH7A1 | KMT2A |
| ALDH7A1 | POLR2A |
| ALDH7A1 | RAD21 |
| ALDH7A1 | SMARCA4 |
| ALDH7A1 | STAT3 |
| ALDH7A1 | TBP |
| ALDH7A1 | TEAD1 |
| ALDH7A1 | TEAD4 |
| ALDH7A1 | TFAP2A |
| ALDH7A1 | YY1 |
| BDNF | CEBPB |
| BDNF | FOS |
| BDNF | FOXA1 |
| BDNF | FOXA2 |
| BDNF | GATA2 |
| BDNF | GATA3 |
| BDNF | HOXB13 |
| BDNF | MYCN |
| BDNF | NANOG |
| DLAT | MAX |
| DLAT | NRF1 |
| DLAT | SPI1 |
| DLAT | TAL1 |
| DLAT | YY1 |
| ECI2 | EGR1 |
| ECI2 | ELF1 |
| ECI2 | EP300 |
| ECI2 | ERG |
| ECI2 | FOS |
| ECI2 | GABPA |
| ECI2 | GATA1 |
| ECI2 | GATA2 |
| ECI2 | HNF4A |
| ECI2 | MAFK |
| ECI2 | MAZ |
| ECI2 | BRD2 |
| ECI2 | RAD21 |
| ECI2 | REST |
| ECI2 | SPI1 |
| ECI2 | STAG1 |
| ECI2 | TAL1 |
| ECI2 | YY1 |
| ECI2 | CEBPB |
| ECI2 | CTCF |
| NDUFA6 | E2F1 |
| NDUFA6 | EGR1 |
| NDUFA6 | FOXA1 |
| NDUFA6 | FOXA2 |
| NDUFA6 | HNF4A |
| NDUFA6 | HOXB13 |
| NDUFA6 | NRF1 |
| NDUFA6 | POLR2A |
| NDUFA6 | RAD21 |
| NDUFA6 | BRD3 |
| NDUFA6 | RUNX1 |
| NDUFA6 | SP1 |
| NDUFA6 | TBP |
| NDUFA6 | YY1 |
| NDUFA6 | CTCF |
| YWHAZ | E2F1 |
| YWHAZ | EGR1 |
| YWHAZ | ELF1 |
| YWHAZ | ERG |
| YWHAZ | ETS1 |
| YWHAZ | ETV1 |
| YWHAZ | FOS |
| YWHAZ | FOSL1 |
| YWHAZ | FOSL2 |
| YWHAZ | FOXA1 |
| YWHAZ | GABPA |
| YWHAZ | ATF1 |
| YWHAZ | JUN |
| YWHAZ | JUND |
| YWHAZ | MAX |
| YWHAZ | NFYA |
| YWHAZ | NFYB |
| YWHAZ | NRF1 |
| YWHAZ | POLR2A |
| YWHAZ | RFX5 |
| YWHAZ | TBP |
| YWHAZ | CREB1 |

***Supplementary 4*. The data of five key genes and 42 miRNAs.**

| miRNA | mRNA |
| --- | --- |
| hsa-miR-150-5p | ACO1 |
| hsa-miR-15a-5p | BDNF |
| hsa-miR-16-5p | BDNF |
| hsa-miR-103a-3p | BDNF |
| hsa-miR-107 | BDNF |
| hsa-miR-182-5p | BDNF |
| hsa-miR-1-3p | BDNF |
| hsa-miR-15b-5p | BDNF |
| hsa-miR-195-5p | BDNF |
| hsa-miR-206 | BDNF |
| hsa-miR-155-5p | BDNF |
| hsa-miR-365a-3p | BDNF |
| hsa-miR-381-3p | BDNF |
| hsa-miR-424-5p | BDNF |
| hsa-miR-410-3p | BDNF |
| hsa-miR-495-3p | BDNF |
| hsa-miR-497-5p | BDNF |
| hsa-miR-613 | BDNF |
| hsa-miR-140-3p | BDNF |
| hsa-miR-300 | BDNF |
| hsa-miR-5688 | BDNF |
| hsa-miR-96-5p | DLAT |
| hsa-miR-365a-3p | DLAT |
| hsa-miR-1271-5p | DLAT |
| hsa-miR-124-3p | ECI2 |
| hsa-miR-30a-5p | YWHAZ |
| hsa-miR-30c-5p | YWHAZ |
| hsa-miR-30d-5p | YWHAZ |
| hsa-miR-30b-5p | YWHAZ |
| hsa-miR-134-5p | YWHAZ |
| hsa-miR-193a-3p | YWHAZ |
| hsa-miR-320a | YWHAZ |
| hsa-miR-30e-5p | YWHAZ |
| hsa-miR-375 | YWHAZ |
| hsa-miR-328-3p | YWHAZ |
| hsa-miR-339-5p | YWHAZ |
| hsa-miR-451a | YWHAZ |
| hsa-miR-193b-3p | YWHAZ |
| hsa-miR-708-5p | YWHAZ |
| hsa-miR-320b | YWHAZ |
| hsa-miR-320c | YWHAZ |
| hsa-miR-320d | YWHAZ |
| hsa-miR-1277-5p | YWHAZ |
